# Can inhaled foreign body mimic asthma in an adolescent?

**DOI:** 10.11604/pamj.2020.36.38.20037

**Published:** 2020-05-27

**Authors:** Alessandro Bodini, Luca Pecoraro, Filippo Catalano, Melodie Olivia Aricò, Laura Tenero, Michele Piazza, Giorgio Piacentini

**Affiliations:** 1 Department of Surgical Sciences, Dentistry, Gynecology and Pediatrics, Pediatric Clinic, University of Verona, Verona, Italy; 2 Emergency Endoscopy Unit, Borgo Trento Hospital, Verona, Italy

**Keywords:** Foreign body aspiration, asthma, differential diagnosis, bronchoscopy, cough, adolescent

## Abstract

A 14 year old male was diagnosed with asthma but didn't improve with appropriate inhalation therapy. Rigid bronchoscopy revealed a food fragment, almost completely occluding the lower-left bronchus lumen. Based on the reported history, it had been likely there for several years.

## Introduction

Inhalation of a foreign body may occur in children especially during the first 3 years of life [1]. It is usually followed by immediate choking, coughing and respiratory distress. Less frequently, it may cause mild, non-specific respiratory symptoms, which may delay the diagnosis and appropriate treatment [2]. In some cases, the diagnostic delay can last years. We describe a case of Foreign Body Aspiration (FBA) misdiagnosed as a difficult asthma in an adolescent.

## Patient and observation

**Figure 1:**
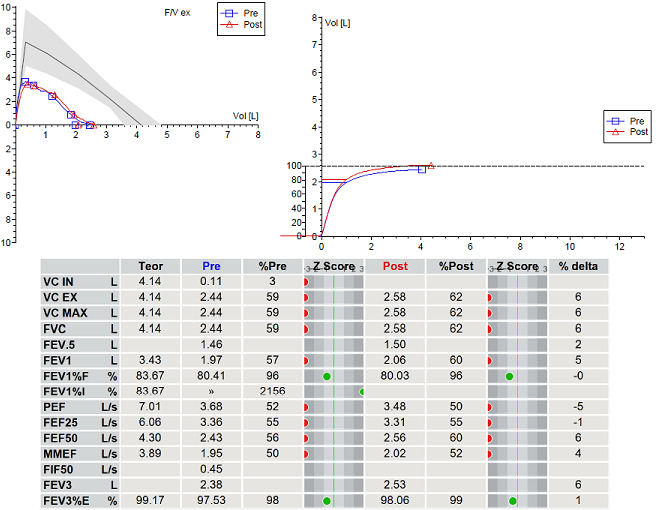
Spirometry before removing the foreign body

**Figure 2:**
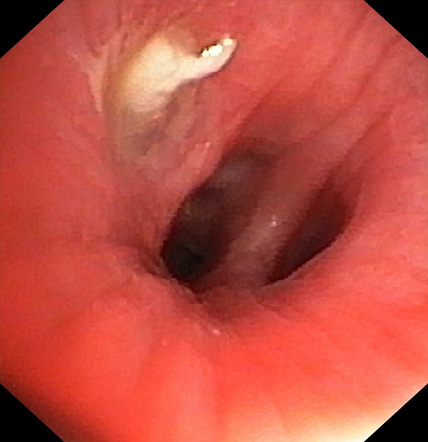
Bronchoscopy image showing the foreign body in the lower-left bronchus

A 14 year old male was diagnosed with asthma but didn't improve with appropriate inhalation therapy. He was obese and also a cigarette smoker, one year before he was admitted for “pneumonia”. Bronchoscopy provided evidence of atelectasis caused by a mucus plug in the lower left bronchus which was removed. On admission, he complained of persistent cough not responding to inhalation therapy with steroids and long acting beta agonist. His skin prick tests were positive for grass; spirometry revealed FEV 1 57% without broncho-reversibility (Figure 1). He was started on inhalation therapy with fluticasone propionate 200mcg/d. Cigarette smoking withdrawal and correction of obesity were proposed. At follow up evaluation 3 months later, he showed no improvement of lung function at spirometry. Thus, inhalation therapy was step-up with fluticasone propionate/salmeterol, at the dose of 25/50mcg (100/200mcg/d) and subsequently at the dose of 25/125mcg (100/500mcg/d), without clinical and functional improvement. Meanwhile, he had continued to smoke and his weight remained stable. Imaging study with chest x-ray and computed tomography (CT) scan showed a new atelectasis engaging the lower-left lung. Therefore, rigid bronchoscopy under general anesthesia was performed and off-white non-purulent secretions, almost completely occluding the bronchial lumen, were visualized and removed from lower-left bronchus (Figure 2). The removed material was analyzed and found to be compatible with a piece of food. Rigid bronchoscopy under general anesthesia was repeated after 3 months: a residual foreign body was still present and was completely removed. Afterwards, chest CT and spirometry were repeated after 2 months for final evaluation and both turned out to be completely normal.

## Discussion

In adolescents with asthma who fail to improve after an appropriate inhalation therapy, further clinical conditions should be taken into account [3]. Specifically, the Global Initiative for Asthma (GINA) suggests a lot of conditions: chronic upper airway cough syndrome, vocal cord dysfunction, dysfunctional breathing, bronchiectasis, cystic fibrosis, congenital heart disease, alpha-1 antitrypsin deficiency, inhaled foreign body [3]. In our case, persistent cough and the absence of improvement of the spirometric scores persisted well beyond the initial improvement, despite first using low inhaled corticosteroid (ICS) and then a higher dose of ICS/Long-Acting Beta-Agonist (LABA), according to step-up options suggested by GINA initiative [3]. An adolescent with a diagnosis of difficult asthma, who doesn't improve although conventional inhalation therapy and finding of unilateral atelectasis and air trapping at chest x-ray, has to be investigated by a bronchoscopy to exclude differential diagnosis of asthma. This case allows to study in deep the role of spirometry and radiology in the diagnosis of asthma and its differential diagnosis. Spirometry has a fundamental role in the assessment of patients with suspected chronic disease of the airway, allowing the distinction between asthma and fixed airflow obstruction [3]. Both spirometric scores and flow-volume (F-V) curve have to be considered. The presence of reduced FEV1/FVC (normally >0,75-0,80 in adults and >0,90 in children) and the absence of reversible airflow limitation are often not compatible with diagnosis of asthma. As a matter of fact, the presence of “variable expiratory airflow limitation” is a cornerstone of the definition of asthma. Anyway, as in the case of our patient, it is not easy to understand the etiology of a chronic airflow limitation in an adolescent: the inhalation therapy is not the only option to treat this condition; all modifiable risk factors, such as smoking and obesity, has to be treated [3].

About F-V curve, it is a graphic plot consisting of inspiratory and expiratory flow against volume obtained while a patient performs maximal forced inspiratory and expiratory maneuvers [4]. The morphology of F-V curve is different between asthma and unilateral main bronchial stenosis (due to a piece of food in the case of our patient). Specifically, the F-V curve in patients with asthma appears as biphasic because of small airway narrowing, which increases the time required to empty the lung. Starting from the evidence that the American Thoracic Society/European Respiratory Society statement considers F-V curve plateau pattern significant only in cases of tracheal stenosis, but doesn't indicate a typical pattern for the F-V curve in cases of a unilateral main bronchial stenosis [5]. Ko *et al.* hypothesized an underlying physiological mechanism to explain them through a similar biphasic F-V curve pattern. Specifically, it is not identifiable during the inspiration phase, but in the expiratory phase, that is composed of two distinct F-V curves: the early expiratory phase is determined by the normal lung and the late phase by the abnormal lung. This event can be explained by positive transmural pressure during the inspiration because the bronchial pressure exceeds the surrounding pleural pressure; consequently, no downstream compression occurs, resulting in greater inspiratory flow. Anyway, in unilateral bronchial stenosis, the shape of the biphasic F-V curve changes with the progression of stenosis because of the migration of the breakpoint; moreover, it can be seen when stenosis is >25% of the lumen of the bronchus [4].

In adolescents with asthma who fail to improve after an appropriate inhalation therapy, radiologic diagnostic investigation is not less important. In the case of our patient, a chest radiography and CT scan were carried out, with a former radiological report of an atelectasis engaging the lower-left lung. This radiologic finding, due to an inhaled foreign body, can be physiopathologically explained: on inhalation as the lung expands, air flows in around the foreign body, but on expiration the object blocks air from getting out causing air trapping. This “ball-valve” mechanism results in hyperlucency and hyperinflation of the involved lung or lobe (with possible tracheal deviation towards the opposite side), which is most evident on a pulmonary x-ray taken at full expiration following a radiography at full inspiration [6]. FBA most frequently occurs in children during the first 3 years of life, with nuts (especially peanuts), seeds, pieces of fruits or vegetables, and small toys being the most common inhaled objects [7]. Most children with FBA presents with acute choking, coughing and respiratory distress. Nevertheless, a negative history does not exclude FBA. If the foreign body moves below to a less critical area of the airways, the initial choking/coughing symptoms settle down or may be missing and the diagnosis is often delayed until complications occur, such as chronic cough, unexplained fever, drug resistant-pneumonia, asthma not-responsive to conventional inhalation therapy, haemoptysis or atelectasis [8]. The first line recommended management of inhaled foreign body in children is prompt removal by rigid bronchoscopy under general anesthesia [7].

## Conclusion

This case emphasizes the need to consider differential diagnosis of a “false” difficult asthma that does not improve with appropriate inhalation therapy. History, spirometry and radiologic diagnostic investigation represents fundamental tools in this evaluation. About FBA, it has to be suspected especially when radiologic findings reveal asymmetrical features. It represents a significant diagnostic challenge for either emergency department and primary care physicians, because it can present with mild non-specific respiratory symptoms, which may lead to delayed diagnosis and inappropriate treatments. It usually occurs in children during the first 3 years of life but is possible in the adolescent age group too. So, it is mandatory to think about it in this age of life.

## Competing interests

The authors declare no competing interests.

## References

[1] Cohen S (2015). Foreign body aspiration in children. Harefuah.

[2] Morice AH, Millqvist E, Bieksiene K, Birring SS, Dicpinigaitis P, Ribas CD (2019). ERS guidelines on the diagnosis and treatment of chronic cough in adults and children. Eur Respir J.

[3] Global Initiative for asthma 2019. Global strategy for asthma management and prevention.

[4] Ko Y, Yoo JG, Yi CA, Lee KS, Jeon K, Um SW (2015). Changes in the flow-volume curve according to the degree of stenosis in patients with unilateral main bronchial stenosis. Clin Exp Otorhinolaryngol.

[5] Pellegrino R, Viegi G, Brusasco V, Crapo RO, Burgos F, Casaburi R (2005). Interpretative strategies for lung function tests. Eur Respir J.

[6] Mortellaro VE, Iqbal C, Fu R, Curtis H, Fike FB, St Peter SD (2013). Predictors of radiolucent foreign body aspiration. J Pediatr Surg.

[7] Wang K, Harnden A, Thomson A (2010). Foreign body inhalation in children. BMJ.

[8] Naviglio S, Chinello M, Ventura A. A (2015). pneumonia that does not improve. Arch Dis Child Educ Pract Ed.

